# *Bacillus licheniformis* PF9 improves barrier function and alleviates inflammatory responses against enterotoxigenic *Escherichia coli* F4 infection in the porcine intestinal epithelial cells

**DOI:** 10.1186/s40104-022-00746-8

**Published:** 2022-07-08

**Authors:** Qiao Li, Linyan Li, Yanhong Chen, Changning Yu, Paula Azevedo, Joshua Gong, Chengbo Yang

**Affiliations:** 1 College of Veterinary Medicine, Nanjing Agricultural University, Nanjing, 210095 China; 2 Department of Animal Science, University of Manitoba, Winnipeg, Manitoba R3T 2N2 Canada; 3 Guelph Research and Development Centre, Agriculture Agri-Food Canada, Guelph, Ontario N1G 5C9 Canada; 4 State Key Laboratory of Food Science and Technology, Nanchang University, Nanchang, 330047 China

**Keywords:** *Bacillus licheniformis* PF9, Barrier function, ETEC F4, Inflammatory response, IPEC-J2

## Abstract

**Background:**

Enterotoxigenic *Escherichia coli* (ETEC) F4 commonly colonizes the small intestine and releases enterotoxins that impair the intestinal barrier function and trigger inflammatory responses. Although *Bacillus licheniformis* (*B. licheniformis*) has been reported to enhance intestinal health, it remains to be seen whether there is a functional role of *B. licheniformis* in intestinal inflammatory response in intestinal porcine epithelial cell line (IPEC-J2) when stimulated with ETEC F4.

**Methods:**

In the present study, the effects of *B. licheniformis* PF9 on the release of pro-inflammation cytokines, cell integrity and nuclear factor-κB (NF-κB) activation were evaluated in ETEC F4-induced IPEC-J2 cells.

**Results:**

*B. licheniformis* PF9 treatment was capable of remarkably attenuating the expression levels of inflammation cytokines tumor necrosis factor-α (TNF-α), interleukin (IL)-8, and IL-6 during ETEC F4 infection. Furthermore, the gene expression of Toll-like receptor 4 (*TLR4*)-mediated upstream related genes of NF-κB signaling pathway has been significantly inhibited. These changes were accompanied by significantly decreased phosphorylation of p65 NF-κB during ETEC F4 infection with *B. licheniformis* PF9 treatment. The immunofluorescence and western blotting analysis revealed that *B. licheniformis* PF9 increased the expression levels of zona occludens 1 (ZO-1) and occludin (OCLN) in ETEC F4-infected IPEC-J2 cells. Meanwhile, the *B. licheniformis* PF9 could alleviate the injury of epithelial barrier function assessed by the trans-epithelial electrical resistance (TEER) and cell permeability assay. Interestingly, *B. licheniformis* PF9 protect IPEC-J2 cells against ETEC F4 infection by decreasing the gene expressions of virulence-related factors (including *luxS*, *estA*, *estB*, and *elt*) in ETEC F4.

**Conclusions:**

Collectively, our results suggest that *B. licheniformis* PF9 might reduce inflammation-related cytokines through blocking the NF-κB signaling pathways. Besides, *B. licheniformis* PF9 displayed a significant role in the enhancement of IPEC-J2 cell integrity.

**Supplementary Information:**

The online version contains supplementary material available at 10.1186/s40104-022-00746-8.

## Background

The intestine is not only a site of nutrient digestion, absorption and metabolism, but also an important defense line against the invasion of pathogenic agents in the external environment of the gut lumen. Intestinal epithelial cells (IECs) are continuous tightly packed monolayer cells lining intestine, which play important roles in this defense [1]. An increase of pathogenic bacteria in IECs can influence intestinal barrier function and change the structure of microbiota, leading to inflammatory reaction [2, 3]. In particular, the disruption of barrier function causes damage to immune homeostasis and increases gut diseases such as inflammatory bowel diseases and diarrhea [3, 4]. Therefore, the maintenance of the normal barrier function of IECs contributes to gut homeostasis and health in humans and animals.

Enterotoxigenic *Escherichia coli* (ETEC) is a common enteric pathogen causing intestinal disorder and diarrhea, which is one of the major challenges for humans and animals, especially in pigs [4, 5]. In humans, the clinical symptoms of ETEC infections can range from mild diarrhea to a severe cholera-like syndrome, which seriously threatens the health of people [6]. In pigs, ETEC-induced diarrhea can reduce growth performance as well as increase morbidity and mortality in post-weaning piglets, resulting in considerable economic losses to the swine industry [7, 8]. The swine industry has largely relied on the prophylactic use of antibiotics to control ETEC and its associated diarrhea in the past [9]. However, there is a growing public interest regarding this practice due to the widespread antibiotic resistance in zoonotic bacterial pathogens, which poses a threat to public health. Furthermore, the World Health Organization has strictly restricted the use of antibiotics in food-producing animals since 2017 [10]. Therefore, non-antibiotic strategies and technologies to control the pathogen using a natural alternative to antibiotics are urgently required to reduce their use in swine production.

The intestinal mucosal barrier plays a major role in maintaining the homeostasis of the gut microenvironment. Porcine intestinal epithelial cells (IPEC-J2) are recognized as a cell model for detecting intestinal inflammation and barrier function [11]. The integrity and regular permeability of tight junctions (TJs) were negatively influenced in IPEC-J2 cells with the invasion of ETEC [12]. Furthermore, ETEC infection could increase the permeability of TJs in early weaned piglets [13]. Probiotics have been defined as living organisms that developed commercially for humans uses, primarily as novel foods or dietary supplements, and in animal feeds for the prevention of gastrointestinal infections, with extensive use in the swine industries [14].

Probiotics have the beneficial effects of maintaining the normal intestinal milieu, modulating the immune system, and producing metabolites required for intestinal health [15]. Specific strains of probiotics have some potential to maintain intestinal barrier function and to reduce cell damage after being impaired by cytokines, chemicals, or pathogens [16]. *Bacillus licheniformis* (*B. licheniformis*), one of the most important bacteria, is used to produce bacitracin against pathogens, ameliorate intestinal flora homeostasis, and improve the nutritional value of animal feeds [17, 18]. Research demonstrated that *B. licheniformis* YB9 significantly repaired the dysbiosis of intestinal flora in mice, characterized by decreasing the abundance of the potentially harmful bacteria *Turicibacter* [17]. Oral administration of a selected mixture of *B. licheniformis* BL1721, *Lactobacillus johnsonii* L531 and *B. subtilis* BS1715 could alleviate inflammation and maintain mucosal barrier integrity in the ileum of pigs challenged with *salmonella* [19]. Another research showed that oral administration of the probiotic mix, i.e., *B. licheniformis* and *B. subtilis* could partially ameliorate *E. coli*-induced enteritis by preventing loss of intestinal epithelial barrier integrity via elevating zonula occludens-1 (ZO-1) expression in weaned pigs [20]. Therefore, it seems that *B. licheniformis* has a great potential application value for preventing pathogenic bacteria infections and maintaining a healthy gut in vivo. However, few research was reported on the effects of *B. licheniformis* on intestinal barrier and inflammation in vitro especially in IPEC-J2. In this study, we hypothesized that the probiotic *B. licheniformis* PF9, which was isolated from feces of piglets, influenced the expression of tight junction proteins and alleviated inflammatory reactions in IPEC-J2 cells that contributed to a protective effect against ETEC F4-induced damage. Therefore, our study aims to explore the protective role of *B. licheniformis* PF9 in improving barrier disruption and inflammatory responses when pathogenic invasion occurs in the intestinal epithelial cells.

## Materials and methods

### Materials

Dulbecco’s modified Eagle’s medium nutrient mix F12 (DMEM/F12), fetal bovine serum (FBS), double-antibiotics (containing 100 U/mL penicillin and 100 U/mL streptomycin), and epidermal growth factor (EGF) were from Invitrogen (Thermo Fisher Scientific, Ottawa, ON, Canada).

### Cell culture

IPEC-J2 cells (ACC 701, RRID: CVCL_2246, DSMZ-German Collection of Microorganisms and Cell Culture, Braunschweig, Germany) were cultured in DMEM/F12 culture medium supplemented with 5% FBS, 1% double-antibiotics and 3 ng/mL EGF in the humidified incubator (Thermo Electron Corporation, Burlington, ON, Canada) with atmosphere of 5% CO_2_ at 37 °C. The culture medium was replaced every 2 days. After growing to 80%–90% confluence, the cells were digested with 0.25% trypsin and inoculated into cell culture plates for further experiments.

### Bacterial strains

The strain of ETEC F4 was isolated from feces of piglets infected with post-weaning diarrhea in the Veterinary Diagnostic Services Laboratory, Government of Manitoba, Canada. The ETEC F4 strain was preserved in the 2-mL cryovials including cryopreservative-added broth. ETEC F4 was first incubated in Tryptic soy broth (TSB, Sigma-Aldrich, Oakville, ON, Canada) at 37 °C for 16–18 h. After dilution with fresh medium, the cultures were incubated under the same condition until the appearance of logarithmic phase. The optical density (OD) value at 600 nm was measured by spectrophotometer (Biochrom™, Thermo Fisher Scientific, Ottawa, ON, Canada). The OD values around 0.3–0.5 were used for the following experiments.

The strain of *B. licheniformis* PF9 was obtained from the Guelph Food Research Centre, Agriculture and Agri-Food Canada and preserved in sterile glycerol (10%–20%). The *B. licheniformis* PF9 strain was isolated from feces of piglets and incubated in TSB at 37 °C overnight (about 16 h). The OD values around 0.8–1.0 were used for the following experiments.

### Experimental design

IPEC-J2 cells were detached with 0.25% trypsin (GibcoTM, Thermo Fisher Scientific, Ottawa, ON, Canada) and diluted to 4 × 10^5^ cells/mL. IPEC-J2 cells were then inoculated into different well plates and cultured in DMEM/F12 medium for about 2–3 d until growing to 80%–90% confluence. For different treatments, the control group was cultured in DMEM/F12 medium without treatment, and the ETEC group was treated with ETEC F4 (with a final concentration of 1 × 10^6^ CFU/mL in DMEM/F12 medium) for 3 h. The ETEC + BL group and the BL group were pretreated with the selected concentrations of *B. licheniformis* PF9 for 2 h. After rinsing in PBS three times, the medium in the ETEC + BL group was then replaced with both ETEC F4 and *B. licheniformis* PF9 (1:5, 1:10, 1:25, 1:50, or 1:100) for another 3 h, and the BL group was then replaced with *B. licheniformis* PF9 alone treatment (3 h).

### Lactate dehydrogenase (LDH) assay

LDH, a cytosolic enzyme present in many different cell types, will be released into the surrounding cell culture media when there is damage to cells. IPEC-J2 cells were detached with 0.25% trypsin and diluted to 4 × 10^5^ cells/mL. IPEC-J2 cells were then inoculated into 24 well plate at 2 × 10^5^ cells/well and cultured in DMEM/F12 medium for 3 d. After the previously described treatments, the supernatants containing released LDH of cultured IPEC-J2 cells were collected to determine the integrity of cells. After centrifugation at 12,000 r/min for 5 min at 4 °C, supernatants were carefully collected and transferred into new tubes. The supernatants of each sample (50 μL) were added into a 96-well plate and the reaction mix for the assay was prepared by using LDH assay kit (CyQUANTTM, Thermo Fisher Scientific, Ottawa, ON, Canada) according to the manufacturer’s instructions. The absorbance at 490 nm and 680 nm was immediately measured by using a Synergy H4 hybrid multi-mode microplate reader (BioTek, Winooski, VT, USA). The activity of LDH was presented as a percentage of the LDH activity by the control group.

### Cytokine measurement by ELISA

The IL-8 concentrations of culture supernatants were measured by ELISA kit (Invitrogen, Thermo Fisher Scientific, Ottawa, ON, Canada) following the manufacturer’s instructions. Briefly, 100 μL of culture supernatant was used for IL-8 assay. At the end of the reaction process, the plates were read at 450 nm, using a Synergy H4 hybrid multi-mode microplate reader (BioTek, Winooski, VT, USA).

### Quantitative PCR (qPCR)

IPEC-J2 cells were seeded with a density of 5 × 10^5^ cells per well in 12-well culture plates. After different treatments, total RNA was extracted by using Trizol (Invitrogen, Carlsbad, CA, USA) according to the manufacturer’s instructions. The total RNA was dissolved in 20 μL RNase-free water and stored at − 80 °C. The RNA concentration was determined using a NanoDrop 2000 Spectrophotometer (Nano-Drop Technologies, Wilmington, DE, USA) with purity ascertained (A260/A280) between 1.8 and 2.0. About 1 μg total RNA from each sample was converted into cDNA by using iScript cDNA Synthesis Kit (Bio-Rad, Mississauga, ON, Canada) according to the manufacturer’s instruction. The primers were designed with Primer-Blast (https://www.ncbi.nlm.nih.gov/tools/primer-blast/index.cgi?LINK_LOC=BlastHome) and synthesized by Integrated DNA Technologies, Inc. The qPCR was performed to quantify the target genes, such as cytokines genes, barrier function genes, toll-like receptors genes, NF-κB signaling pathway genes, and virulence-related genes (Additional file 1: Tables S1 and S2). The cyclophilin-A (*CYCA*) gene was used as the housekeeping gene in IPEC-J2 cells. The glyceraldehyde-3-phosphate dehydrogenase A (*GAPA*) gene was used as the housekeeping gene in ETEC F4. The relative changes in gene expression levels in IPEC-J2 cells or ETEC F4 normalized against *CYCA* or *GAPA* were determined by using the 2^−∆∆CT^ method [21].

### Immunofluorescent staining

Cells cultured onto cover-slips (Thermo Fisher Scientific, Ottawa, ON, Canada) were fixed with 4% paraformaldehyde for 20 min and permeabilized with 0.3% Triton X-100 in PBS for 20 min. The cells were blocked with 5% goat serum (Jackson ImmunoResearch Laboratories, West Grove, PA, USA) for 1 h and then incubated with anti-rabbit polyclonal antibodies of zona occludens 1 (ZO-1, 1:100 dilution in PBS) and occludin (OCLN, 1:100 dilution, Thermo Fisher Scientific, Ottawa, ON, Canada) at 4 °C for 16 h, respectively. The cells were then washed 3 times with PBS and incubated with an Alexa Fluor 488 goat anti-rabbit (green) or Alexa Fluor 584 goat anti-rabbit (red) antibody (Catalogue no. A-11034 or A-11037, Thermo Fisher Scientific, Ottawa, ON, Canada) for 1 h at 22 °C. For F-actin staining, the cells were incubated with phalloidin, CF488A (1:40 dilution in PBS, Catalogue no. 94538, Biotium, Inc., Fremont, CA, USA) at 22 °C for 1 h. Rinsed cells were mounted with Vectashield mounting medium with 4,6-Diamidino-2-phenylindole (DAPI, Vector Laboratories, Inc. Burlingame, CA, USA). Images were taken by a Zeiss fluorescence microscope (Car-Zeiss Ltd., Toronto, ON, Canada).

### Transepithelial electric resistance (TEER) and cell permeability measurement

The IPEC-J2 cells with a density of 5 × 10^5^ cells per well were seeded into Millicell membrane cell inserts (12 wells, Corning Costar, New York, NY, USA). The TEER of cell monolayers was measured using a Millicell Electrical Resistance System (ESR-2, Sigma-Aldrich, Oakville, ON, Canada). The TEER was monitored every 2 days until a monolayer of cells was completely differentiated. The TEER of four treatment groups (control, ETEC F4 alone, ETEC F4 plus *B. licheniformis* PF9, and *B. licheniformis* PF9 alone) was measured before (0 h) and after ETEC F4 treatments (1, 2, 3, 4, and 5 h), respectively. The TEER of monolayers with/without adding ETEC F4 represented the ETEC F4 group and control group, respectively. The ETEC F4 plus *B. licheniformis *PF9 group were pre-treated with *B. licheniformis* PF9 for 2 h and then incubated with ETEC F4 plus *B. licheniformis* PF9 for 3 h. The value of TEER was corrected for background resistance by subtracting the contribution of cell free filter and the medium (200 Ω).

To quantify the paracellular permeability of cell monolayers, 0.5 mg/mL of 4 kDa FITC-dextran (FD4, Sigma-Aldrich, Oakville, ON, Canada) was added to the apical side of the inserts. The basolateral medium aliquots were taken after 48 h of incubation. The diffused fluorescent tracer was then measured by fluorometry (excitation, 485 nm, emission, 528 nm) using a microplate reader (BioTek, Winooski, VT, USA). Measurements were performed in quadruplicate.

### Western blotting

Proteins from IPEC-J2 were extracted by using RIPA Lysate Buffer (Sigma-Aldrich, Oakville, ON, Canada) and their concentrations were determined by the BCA Protein Assay Kit according to the manufacturer’s protocol (Thermo Fisher Scientific, Ottawa, ON, Canada). Proteins were resolved by SDS-PAGE and transferred to a PVDF membrane using a Trans-Blot® Turbo™ transfer system (Bio-Rad, Mississauga, ON, Canada). The membrane was then blocked for 2 h with 5% skim milk in Tris-buffered saline (TBS) at 22 °C, and was subsequently immunolabeled in primary antibodies diluted in TBS at 4 °C for 16 h. Primary antibodies to rabbit polyclonal anti-ZO-1 (1:1000 dilution), anti-OCLN (1:1000 dilution), anti-claudin-3 (1:1000 dilution) and anti-NF-κB p65 (1:1000 dilution), rabbit monoclonal anti-NF-κB phospho-p65 (1:1000 dilution) and mouse monoclonal anti-β-actin (1:5000 dilution) from Invitrogen by Thermo Fisher Scientific were used, respectively. After washing for 3 × 10 min with TBST, the blots were incubated with horseradish peroxidase-conjugated goat anti-mouse (1:350 dilution) or goat anti-rabbit IgG (1:4000 dilution) as a secondary antibody for 1 h at 22 °C, respectively. Visualization of the antigen-antibody complex was conducted with a Clarity Max ECL Western Blotting Substrate (Bio-Rad, Mississauga, ON, Canada) and immunoreactive proteins were visualized using the ChemiDoc™ MP imaging system (2.4.0.03, Bio-Rad, Mississauga, ON, Canada). The protein bands were analyzed by Image Lab 6.0 software. The β-actin was used as the internal control. Values of target proteins were represented as the ratio of the optical density of the protein bands to the density of the respective β-actin band. The value of the normalized phosphorylation of p65 protein was represented as the ratio of the density of phosphor-p65 to that of p65.

### Statistical analysis

Data were presented as means ± standard deviations (SD). Statistical analysis was performed using GraphPad Prism 7 (GraphPad Software, La Jolla, CA, USA). Differences between means were evaluated by one-way ANOVA. Tukey’s multiple comparisons test was used. Difference in the mRNA abundance of virulence-related factors in ETEC F4 between ETEC and ETEC+BL groups was evaluated by unpaired *t*-test. The level of significance was set at *P* < 0.05.

## Results

### *B. licheniformis* PF9 protected IPEC-J2 cells against the ETEC F4-induced cytotoxicity

**Fig. 1:**
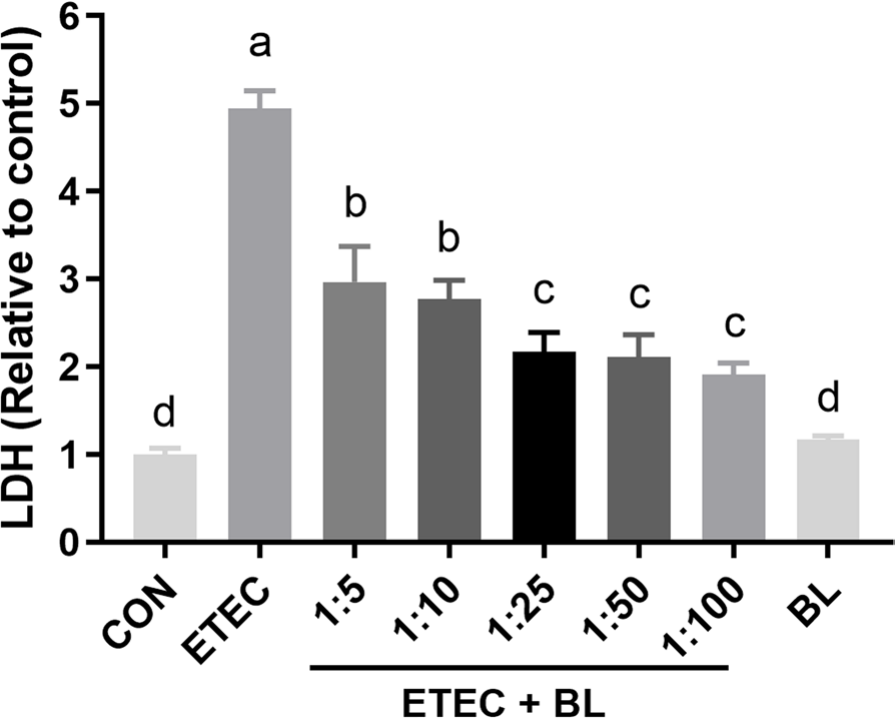
*B. licheniformis* PF9 protected IPEC-J2 cells against the ETEC F4-induced cytotoxicity. LDH activity in the supernatant was detected by LDH detection kit. The CON group was with no treatment. The groups of ETEC and BL were treated with 10^6^ CFU/mL ETEC F4 alone and 10^8^ CFU/mL *B. licheniformis* PF9 alone for 3 h, respectively. The groups of ETEC + BL were treated with both ETEC F4 (10^6^ CFU/mL) and *B. licheniformis* PF9 at the ratio of 1:5, 1:10, 1:25, 1:50, or 1:100 (i.e., 5 × 10^6^, 10^7^, 2.5 × 10^7^, 5 × 10^7^, or 10^8^ CFU/mL) for 3 h, respectively. The data were presented as mean ± SD, *n* = 4. Different letters represent a significant difference (*P* < 0.05)

To quantify the effect of *B. licheniformis* PF9 protected IPEC-J2 cells against the ETEC F4, the cytotoxicity was measured by LDH activity in the supernatants. As shown in Fig. 1, the ETEC group showed that ETEC F4 challenge at 10^6^ CFU/mL significantly induced the toxicity effect in IPEC-J2 cells (*P* < 0.05). However, this cytotoxicity could be remarkably attenuated in the ETEC + BL groups, in which, ETEC F4 was simultaneously added with *B. licheniformis* PF9 at the ratios of 1:5, 1:10, 1:25, 1:50, or 1:100 when compared with that in the ETEC group (*P* < 0.05). Meanwhile, the result of the BL group demonstrated that the cells treated with the highest concentration of *B. licheniformis* PF9 (10^8^) did not significantly increase LDH release compared with control group (*P* > 0.05). These results indicated that the treatment with *B. licheniformis* PF9 maintains the integrity of the cellular membrane in IPEC-J2 cells.

### Effect of *B. licheniformis* PF9 on ETEC F4-induced the alteration of cytokine IL-8 secretion in IPEC-J2 cells

**Fig. 2:**
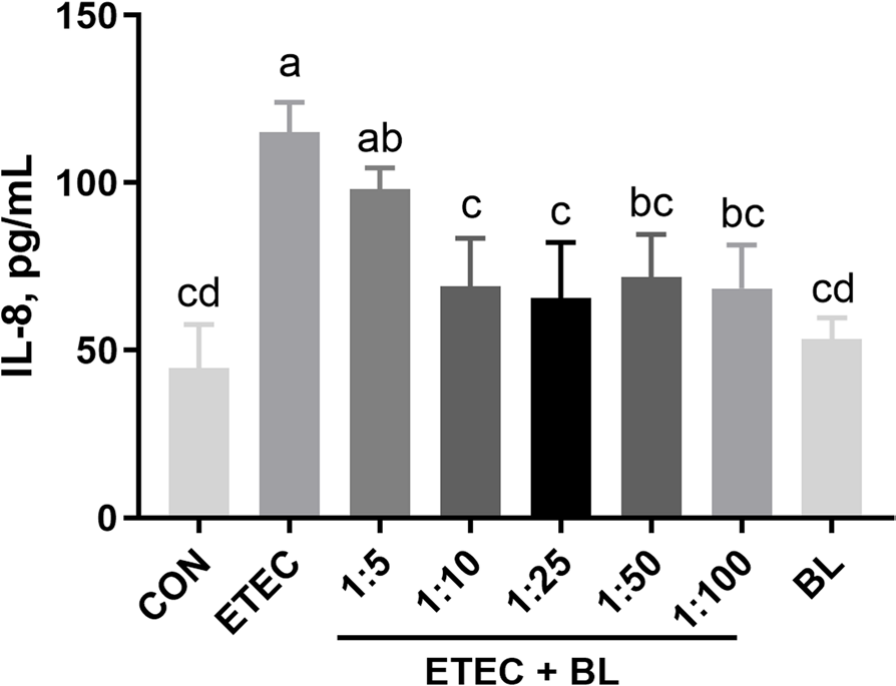
Effect of *B. licheniformis* PF9 on ETEC F4-induced the alteration of cytokine IL-8 secretion in IPEC-J2 cells. The treatments of these groups are the same as the above in Fig. 1. The IL-8 concentration in the supernatant was measured by ELISA assay. The data were presented as mean ± SD, *n* = 4. Different letters represent a significant difference (*P* < 0.05)

The effects of *B. licheniformis* PF9 on cytokine IL-8 secretion changes in IPEC-J2 cells challenged with ETEC F4 were detected by ELISA. As shown in Fig. 2, the result of the BL group demonstrated that the cells treated with the highest concentration of *B. licheniformis* PF9 (10^8^) had no effect on the IL-8 secretion compared with control group (*P* > 0.05). However, the ETEC group showed that ETEC F4 challenge at 10^6^ CFU/mL significantly stimulated the IL-8 secretion in IPEC-J2 cells (*P* < 0.05). Interestingly, this change could be markedly diminished under the treatment of adding both ETEC F4 and *B. licheniformis* PF9 at the ratios of 1:5, 1:10, 1:25, 1:50, or 1:100 (*P* < 0.05). However, this effect seemed not significant with the higher ratios of ETEC F4 and *B. licheniformis* PF9, i.e., dilutions ratios over 1:10*.* Therefore, we chose the ratio of ETEC F4 and *B. licheniformis* PF9 at 1:10 (i.e., 10^6^ CFU/mL ETEC F4:10^7^ CFU/mL *B. licheniformis* PF9) in the following experiment to explore the effects of *B. licheniformis* PF9 against ETEC F4-induced barrier function disruption and inflammatory responses in the IPEC-J2 cells.

### Effect of *B. licheniformis* PF9 on ETEC F4-induced gene expression of inflammatory cytokines

**Fig. 3:**
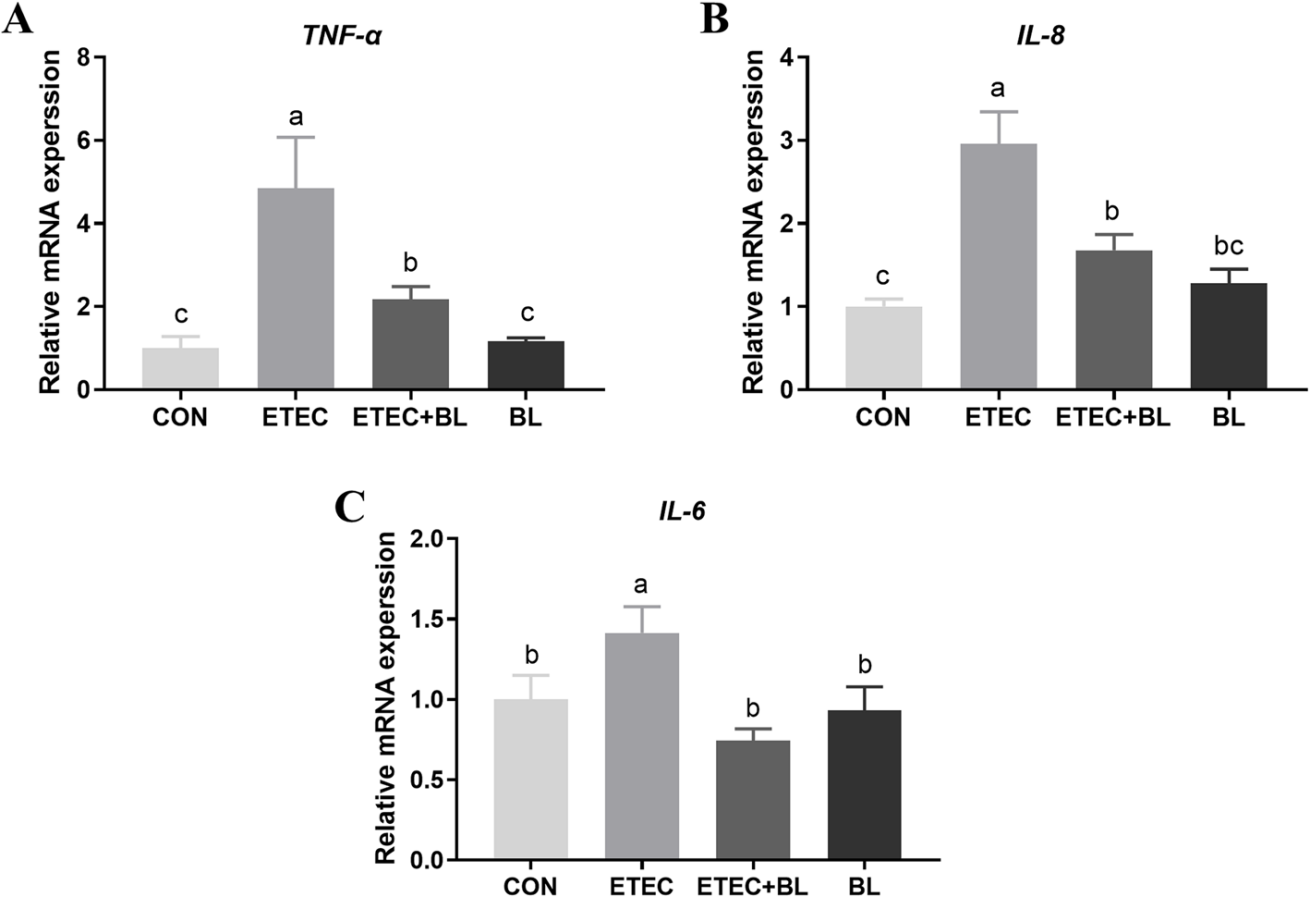
Effect of *B. licheniformis* PF9 on ETEC F4-induced gene expression of inflammatory cytokines in IPEC-J2 cells. The CON group was with no treatment. The groups of ETEC, ETEC + BL, and BL were treated with 10^6^ CFU/mL ETEC F4 alone, both ETEC F4 (10^6^ CFU/mL) and *B. licheniformis* PF9 (10^7^ CFU/mL), and 10^7^ CFU/mL *B. licheniformis* PF9 alone for 3 h, respectively. These treatment groups were also used in the following Figures. Total RNA was extracted from IPEC-J2 cells and the mRNAs abundance of *TNF-α* (**A**), *IL-8* (**B**), and *IL-6* (**C**) were detected by qPCR. The data were presented as mean ± SD, *n* = 4. Different letters represent a significant difference (*P* < 0.05)

In order to investigate the effects of *B. licheniformis* PF9 on expression profiles of inflammatory cytokines on IPEC-J2 cells subjected to ETEC F4 challenge, the mRNA expressions of pro-inflammatory cytokines (*TNF-α*, *IL-8*, and *IL-6*) were followed by qPCR. As shown in Fig. 3, it was demonstrated that *B. licheniformis* PF9 (10^7^ CFU/mL) alone didn’t show differences on the mRNA expression of *TNF-α*, *IL-8*, and *IL-6* when compared with the control group (*P* > 0.05). However, it was shown that ETEC F4 challenge at 10^6^ CFU/mL significantly stimulated the inflammatory gene expressions of *TNF-α*, *IL-8*, and *IL-6* (*P* < 0.05). It is worth noticing that these up-regulations could be markedly decreased under the treatment of adding both ETEC F4 and *B. licheniformis* PF9 at the ratio of 1:10 (*P* < 0.05). Nevertheless, the gene expressions of *TNF-α* and *IL-8* in the ETEC + BL group were still significantly higher than those in the control group (Fig. 3A and B, *P* < 0.05). The gene expressions of IL-6 in the ETEC + BL group showed no difference than those in the control group (Fig. 3C, *P* < 0.05).

### Protective effect of *B. licheniformis* PF9 against ETEC F4-induced morphological changes of cytoskeleton and tight junctions (TJs) in IPEC-J2 cells

**Fig. 4:**
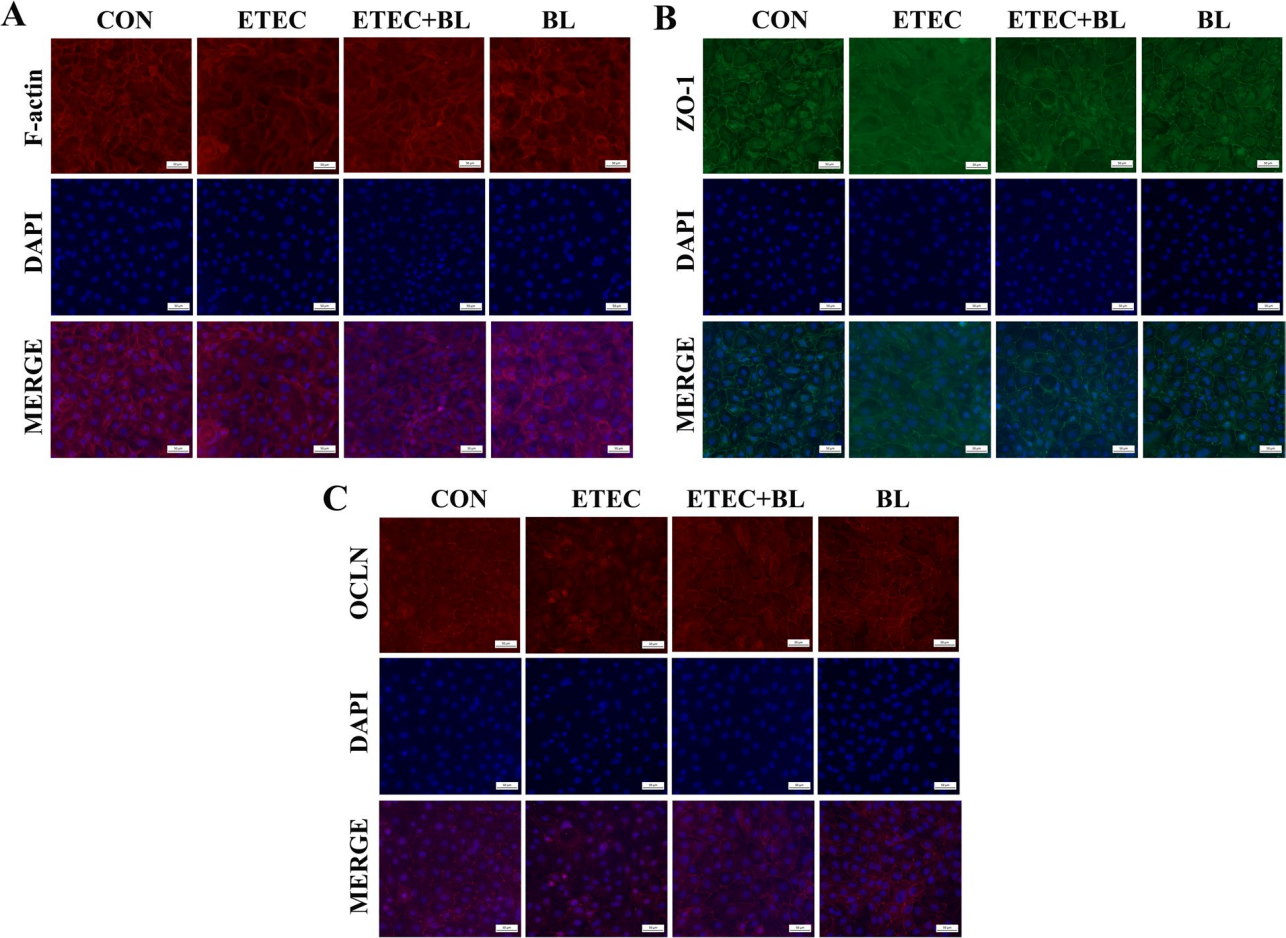
Protective effect of *B. licheniformis* PF9 against ETEC F4-induced morphological changes of cytoskeleton (F-actin, **A**) and TJs (ZO-1 and OCLN, **B** and **C**) in IPEC-J2 cells

The effects of *B. licheniformis* PF9 and pathogen ETEC F4 on the distribution of cytoskeleton (F-actin) as well as TJs (zonula occludes 1, ZO-1 and occludin, OCLN) were evaluated by immunofluorescence. *B. licheniformis* PF9 treatment alone did not alter the expression of cytoskeleton F-actin. The cell morphology displayed disordered and the fluorescence of F-actin seemed to be unclear and even disappeared in some cells after ETEC F4 infection. In comparison, the *B. licheniformis* PF9 treatment notably maintained normal morphology and increased the green fluorescent spots against pathogenic infection in the cytoskeleton of F-actin (Fig. 4A). Meanwhile, there were highly similar morphological changes of TJs proteins. TJs proteins of ZO-1 and OCLN did not show the obvious change after *B. licheniformis* PF9 treatment alone, which were present at the apical intercellular borders in a belt-like manner, encircling the cells and delineating the cellular borders. The fluorescence was discontinuous and unclear in cells infected with ETEC F4. However, *B. licheniformis* PF9 was able to ameliorate the abnormal morphology of junction proteins, whereas the green (ZO-1) or red (OCLN) spots were increased compared with ETEC F4 infection (Fig. 4B and C).

### Influence of *B. licheniformis* PF9 on ETEC F4-induced gene and protein expressions of tight junctions (TJs) in IPEC-J2 cells

**Fig. 5:**
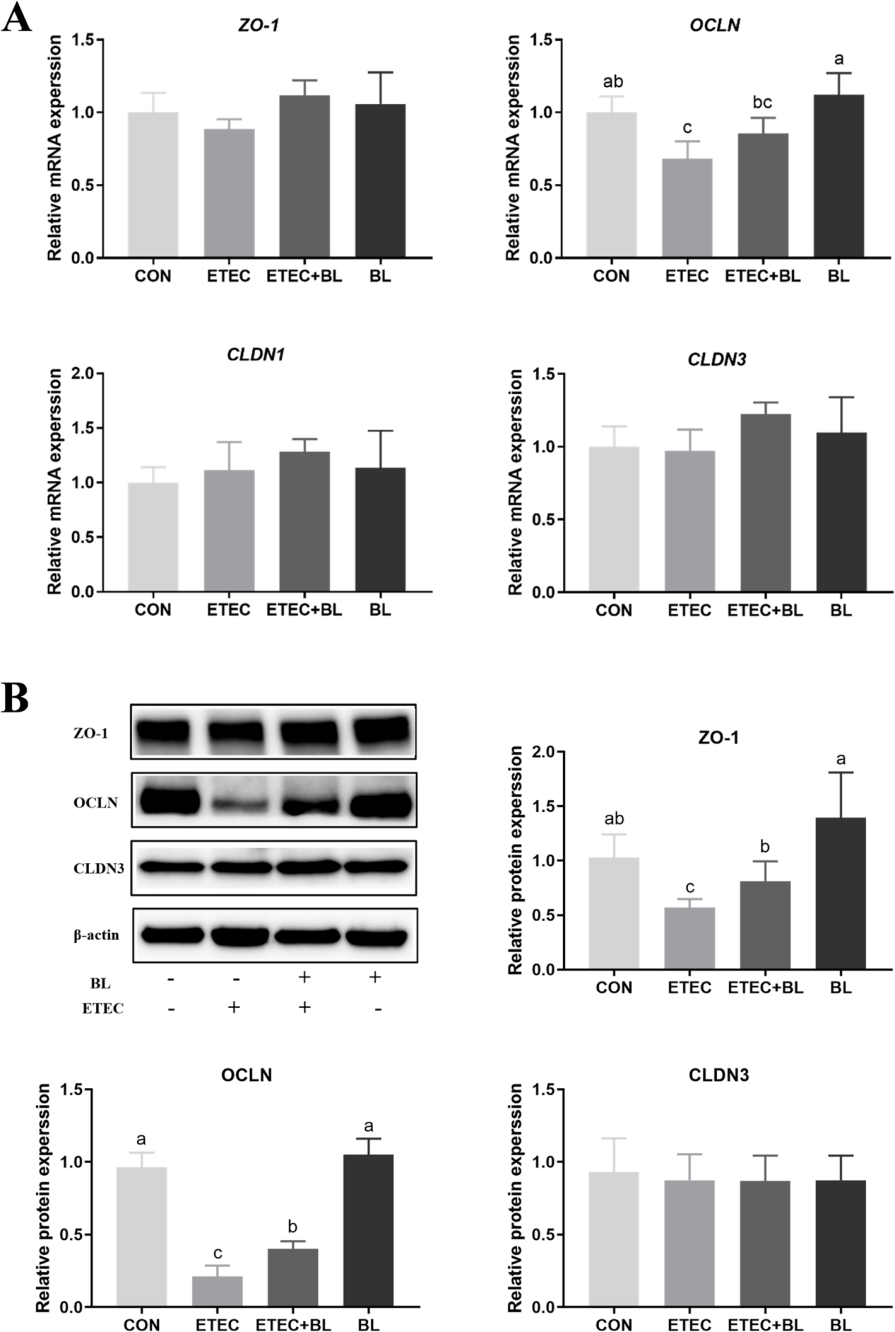
Influence of *B. licheniformis* PF9 on ETEC F4-induced gene and protein expressions of tight junctions (TJs) in IPEC-J2 cells. **A** Total RNA was extracted from IPEC-J2 cells and the gene expressions of *ZO-1*, *OCLN*, *CLDN1*, and *CLDN3* were detected by qPCR (*n* = 4). **B** Total protein was extracted from IPEC-J2 cells and the protein expressions of ZO-1, OCLN, and CLDN3 were detected by western blotting (*n* = 3). The data were presented as mean ± SD. Different letters represent a significant difference (*P* < 0.05)

To investigate the possible membrane barrier disruption caused by ETEC F4 and the potential role of *B. licheniformis* PF9 in restoring such damage, the gene and protein expressions of TJs were performed (Fig. 5). The effects of treatment on the abundance of transcripts of *ZO-1*, *OCLN*, claudin-1 (*CLDN1*) and claudin-3 (*CLDN3*) were examined in IPEC-J2 cells. There were no significant differences in the relative abundance of three transcripts (*ZO-1*, *CLDN1* and *CLDN3*) in the four different treatments (Fig. 5A, *P* > 0.05). However, the abundance of mRNA *OCLN* was significantly reduced after challenge with ETEC F4 (*P* < 0.05), and the treatment with *B. licheniformis* PF9 could reverse this change when compared with the control cells.

Western blotting was used to verify treatment differences in expression of TJ components (ZO-1, OCLN and CLDN3) at the protein level in IPEC-J2 cells (Fig. 5B). From the results, it was found that *B. licheniformis* PF9 did not influence the proteins expressions of ZO-1, OCLN and CLDN3. Meanwhile, in agreement with the mRNA expression of *CLDN3*, there were no changes of protein abundance in the four different groups (Fig. 5B, *P* > 0.05). However, ETEC F4 challenge was able to obviously decrease expression levels of ZO-1 and OCLN in IPEC-J2 cells (*P* < 0.05) while concurrent treatment ETEC F4 with *B. licheniformis* PF9 had the capability to significantly increase the levels of these two TJ proteins (Fig. 5B, *P* < 0.05).

### Protective effect of *B. licheniformis* PF9 against ETEC F4-induced damage to barrier integrity

**Fig. 6:**
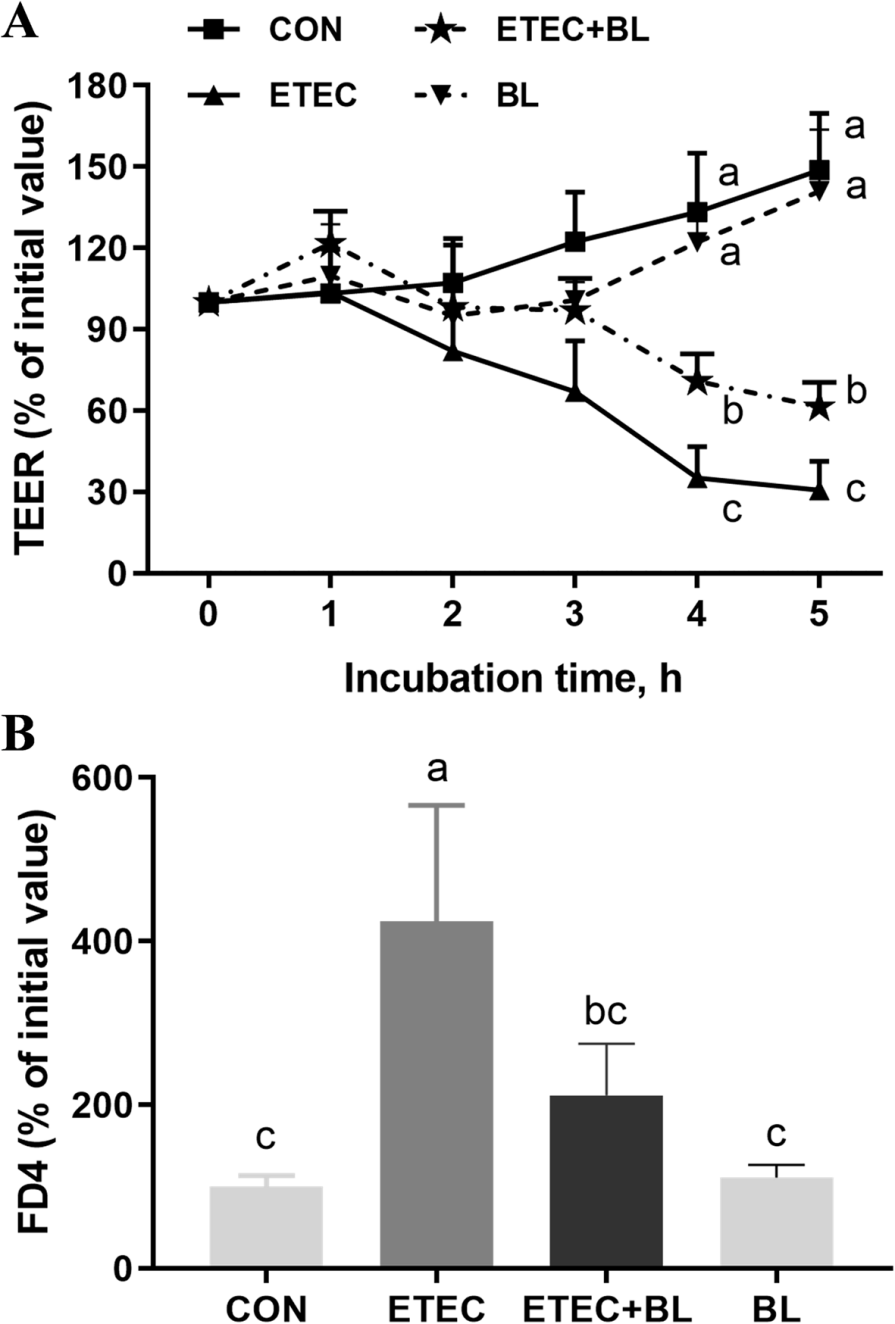
Effect of *B. licheniformis* PF9 on TEER and permeability in ETEC F4-challenged IPEC-J2 cells. **A** These TEER values were measured before (0 h) and after ETEC F4 treatments (1, 2, 3, 4, and 5 h). **B** Cell permeability was tested by the fluorescent intensity of FD4 after *B. licheniformis *PF9 treatment for 5 h. The data were presented as mean ± SD, *n* = 4. Different letters indicate a significant difference at *P* < 0.05

The epithelial barrier function of IPEC-J2 cells monolayer was assessed by TEER and cell permeability in response to ETEC F4 infection in the absence or presence of *B. licheniformis* PF9. As shown in Fig. 6A, single *B. licheniformis* PF9 treatment had no negative effect on the TEER in IPEC-J2 cells, however, the percentage of relative TEER values significant decreased (about 65% and 70%, respectively) in cells infected with ETEC F4 at 4 h and 5 h (*P* < 0.05). When cells were pre-treated with *B. licheniformis* PF9 followed by challenge with ETEC F4, however, the reductions of TEER were less (about 30% and 40%, respectively) at 4 h and 5 h. Consistently, a significant augmentation of fluorescence intensity observed in the ETEC F4 challenge was substantially minimized after *B. licheniformis* PF9 supplementation (Fig. 6B, *P* < 0.05). These results indicated that pre-treatment with *B. licheniformis* PF9, while itself having no disadvantageous effect on the barrier integrity of IPEC-J2 cells, did confer some protection on barrier integrity when cells were subsequently challenged with ETEC F4.

### Effect of *B. licheniformis* PF9 on the mRNA abundance of virulence-related factors in ETEC F4

**Fig. 7:**
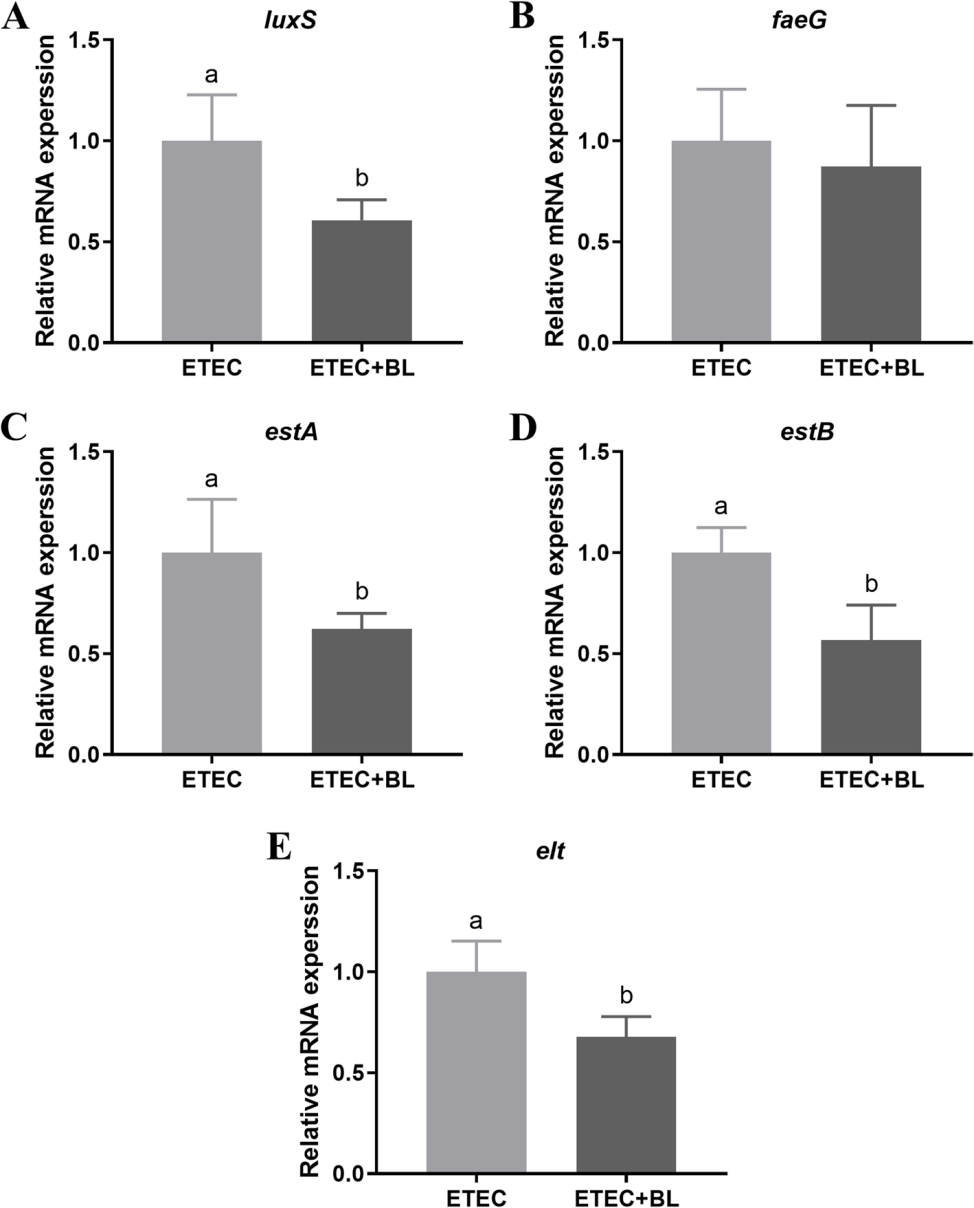
Effect of *B. licheniformis* PF9 on the mRNA abundance of virulence-related factors in ETEC F4. Total RNA was extracted from ETEC F4 and the mRNAs abundance of *luxS* (**A**), *faeG* (**B**), *estA* (**C**), *estB* (**D**), and *elt* (**E**) were detected by qPCR. The data were presented as mean ± SD, *n* = 4. Different letters represent a significant difference (*P* < 0.05)

To determine the effects of *B. licheniformis* PF9 in protecting IPEC-J2 cells against ETEC F4 infection, the gene expressions of virulence-related factors (including *faeG*, *luxS*, *estA*, *estB*, and *elt*) of ETEC F4 were examined in the presence and absence of *B. licheniformis* PF9. The quantitative PCR analysis revealed that co-cultured with *B. licheniformis* PF9 was able to downregulate the expression of bacterial genes related to quorum sensing (*luxS*), as well as STs (*estA* and *estB*) and LT (*elt*) toxin secretion of ETEC F4 in infected IPEC-J2 cells (Fig. 7A, and C-E, *P* < 0.05). However, the *faeG* gene expressions related to fimbrial biosynthesis of ETEC F4 strains did not show obvious difference in the two groups (Fig. 7B, *P* > 0.05). These results suggest that *B. licheniformis* PF9 was involved in alleviating ETEC F4-induced cytotoxicity by decreasing the expressions of virulence-related factors in IPEC-J2 cells.

### The functions of NF-κB involved in TLRs-triggered signaling pathways during the process of *B. licheniformis* PF9 regulating the inflammatory response

**Fig. 8:**
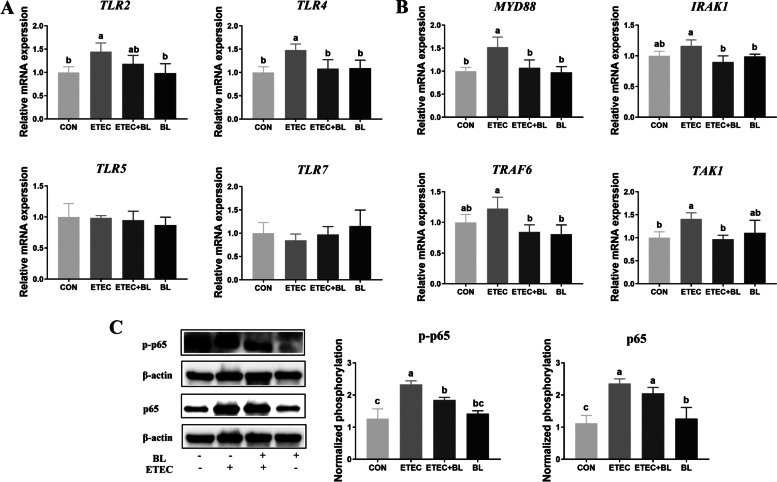
Effect of *B. licheniformis* PF9 on TLRs-mediated activation of the NF-κB signaling pathway after ETEC F4 stimulation in IPEC-J2 Cells. **A** The mRNA abundance of toll-like receptors was detected by qPCR (*n* = 4). **B** The mRNA abundance of the related upstream genes of the NF-κB signaling pathway was detected by qPCR (*n* = 4). **C** The protein abundances of p65 and p-p65 were detected by western blotting (*n* = 3). The data were presented as mean ± SD. Different letters represent a significant difference (*P* < 0.05)

The mRNA expression of toll-like receptors (*TLR2*, *TLR4*, *TLR5*, and *TLR7*) in ETEC F4 were measured by RT-PCR. The gene expression of *TLR2* and *TLR4* was significantly higher (*P* < 0.05) in the ETEC F4 group, but this increase in gene expression could be attenuated by the *B. licheniformis* PF9 treatment. On the other hand, the levels of *TLR5* and *TLR7* mRNAs did not change in the ETEC group compared to the control group (Fig. 8A, *P* > 0.05). Furthermore, compared with the control group, the ETEC F4 increased the mRNA expressions of the related upstream genes of the NF-κB signaling pathway (*MyD88* and *TAK1*). While the mRNA levels of *TRAF6* and *IRAK* did not show significant increase in the ECET F4-induced cells. Meanwhile, *B. licheniformis* PF9 treatment significantly blocked the increase in *MyD88*, *TRAF6*, *IRAK* and *TAK1* mRNA levels (Fig. 8B, *P*< 0.05). The contents of total p65 and phosphorylated p65 protein and increased significantly after ETEC F4 treatment, while *B. licheniformis* PF9 could reverse the increasing phosphorylated p65 protein (Fig. 8C, *P*< 0.05).

## Discussion

The intestine and associated epithelium not only can effectively digest and absorb nutrients, but it is also armed with a large number of immune cells ready to intervene when pathogenic bacteria breach the intestinal epithelium. Gut disorders and dysfunctions might induce gut diseases such as inflammatory bowel diseases and diarrhea, leading to harmful effect to animal health and growth performance [22]. In fact, the intestinal epithelial cells (IECs) play pivotal roles in the physical defense [23]. The maintenance of the barrier function of IECs contributes to gut homeostasis and health. Therefore, nutritional and/or pharmacological interventions for reinforcing this barrier for humans and animal health are gaining much interest.

ETEC is a common enteric pathogen causes colibacillosis resulting in intestinal disorder and diarrhea, which is one of the major challenges for humans and several species of farm animals [6, 24]. ETEC are characterized by producing virulence factors such as adhesins and enterotoxins. The most common adhesins that promote binding and colonization of the intestinal epithelium are expressed in the fimbriae, such as the F4 (also designated K88), F5 (K99), F6 (987P), F17 and F18. Once established in the animal small intestine, ETEC produces enterotoxins, namely labile toxins (LTs) and stable toxins (STs), acting on enterocytes and resulting in diarrhea [25]. In the livestock industry, where the prophylactic use of antibiotics in ETEC infections is being restricted in most countries, an increasing number of antibiotic alternatives are being developed to alleviate intestinal inflammation and protect digestive health in the gut [26]. Probiotics, an attractive group of such alternatives, can provide beneficial health effects to the host by excluding pathogenic bacteria, producing bactericidal agents, regulating immune response and maintaining gut barrier integrity [27, 28]. *B. licheniformis*, Gram-positive bacteria, has been popularly considered as a beneficial probiotic bacterium [29]. A recent study showed that *B. licheniformis* CK1 could alleviate the toxic effects of zearalenone in feed on weaned female Tibetan piglets [30]. The ileum microbiota disorder caused by necrotic enteritis was normalized by dietary *B. licheniformis* supplementation in chickens [31]. Recent research also showed the combination of *B. subtilis *B21 and *B. licheniformis* B26 improved the intestinal health and mucosal barrier of broiler chickens by the regulation of tight junction proteins in chickens with *Clostridium perfringens*-induced necrotic enteritis [32]. Meanwhile, oral administration of *B. licheniformis* and *B. subtilis* mixture ameliorated ETEC F4-induced enteritis in the weaned F4ab/acR^−^ (ETEC F4ab/ac receptor negtive) pigs [33]. Furthermore, *B. licheniformis* was found to have its anti-inflammatory actions by significantly intervening with the secretion of IL-8 in IPEC-J2 [34]. Although *B. licheniformis* strain is especially popular used as an additive in processed feed, the mode of action to protect the gut health from ETEC-induced disruption is not well-understood. In the present study, ETEC F4 bacteria, isolated from feces of piglets infected with post-weaning diarrhea in the Veterinary Diagnostic Services Laboratory from the Government of Manitoba in Canada, was used to establish the model of harmful bacteria invasion in vitro. A previous study from this laboratory, showed that this strain of ETEC F4 can affect the nutrient absorption, immunity and gut barrier function in weaned piglets [35]. It was hypothesized that *B. licheniformis* PF9 isolated from feces of piglets in this study may be involved in attenuating/ameliorating ETEC-induced changes in mucosal barrier function and the inflammatory responses of intestinal epithelial cells.

LDH, an oxidoreductase enzyme, is found in the cytoplasm of many different cells. Previous research showed that both intracellular and extracellular stress can lead to an impaired mucosal barrier function and subsequently stimulate the release of LDH from cells [36]. Thus, the amount of LDH detected in the culture medium is generally used as an indicator for determining damaged cells. In the present study, we found that ETEC F4 challenge at 10^6^ CFU/mL was able to induce a significant increase in LDH release by IPEC-J2 cells. It’s noteworthy to notice though that the addition of both ETEC F4 and *B. licheniformis* PF9 together at the ratios of 1:5, 1:10, 1:25, 1:50, or 1:100 to the culture medium markedly decreased LDH release (Fig. 1), which confirmed a positive role of *B. licheniformis* PF9 in protecting the integrity of the cellular membrane in IPEC-J2 cells.

Cytokine production is considered as an important indicator in response to ETEC F4. Many *B. licheniformis* strains were proved to have the ability to alleviate inflammatory response in vitro and in vivo. One study has demonstrated that *B. licheniformis* strain can significantly decrease the production of the pro-inflammatory cytokines (IL-8) under the challenge of *Salmonella enterica* serovar Typhimurium in intestinal epithelial cells [34]. Pretreatment with *Bacillus* species spores (*B. licheniformis, B. indicus, B. subtilis, B. clausii,* and *B. coagulans*) lowered the serum levels of pro-inflammatory cytokines (TNF-α and IL-1β) against acetaminophen-induced acute liver injury in rats [37]. Similarly, we found that *B. licheniformis* PF9 treated with pathogenic exposure at the ratios of 1:10, 1:25, 1:50, or 1:100 (ETEC F4 to *B. licheniformis* PF9) actually attenuated the release of cytokines IL-8 (Fig. 2). However, this anti-inflammatory effect seemed not to cause any significant change with the higher ratio of ETEC F4 and *B. licheniformis* PF9 (over 1:10). Therefore, we ultimately chose the treatment with the ratio of ETEC F4 and *B. licheniformis* PF9 at 1:10 (i.e., 10^6^ CFU/mL ETEC F4:10^7^ CFU/mL *B. licheniformis* PF9) in the following experiment to explore the effect of *Bacillus licheniformis* PF9 against ETEC F4-induced barrier function disruption and inflammatory responses in the IPEC-J2 cells. Interestingly, it was shown that ETEC F4 challenge significantly stimulated the inflammatory gene expressions of *TNF-α*, *IL-8*, and *IL-6*, which can be markedly decreased under the treatment of adding both ETEC F4 and *B. licheniformis* PF9 at the ratio of 1:10 (Fig. 3)*.* These results suggest that *B. licheniformis* PF9 could down-regulate cytokines production, and probably slow down cell damage and relatively weakening inflammation.

The intestinal barrier plays a vital role in organ development and disease pathogenesis. It is closely associated with functional TJs which are a dynamic complex locating between epithelial cells and play a key role in critical structure for paracellular permeability [38]. In this sense, alteration of this multi-functional and continuous assembled cells layer may contribute to disordering the normal paracellular permeability (ion, water and solutes transport) and epithelium integrity [39]. Occludin (OCLN), ZO-1 and claudins (CLDN1 and CLDN3) are crucial transmembrane proteins to maintain the physiological functions of TJs. Decreasing occludin and ZO-1 expression could increase the intestinal permeability in weaned piglets or IPEC-J2 [11, 40]. The integrity and regular permeability of TJs were negatively influenced by invasion of ETEC F4 in IPEC-J2 cells [12]. Meanwhile, ETEC F4 infection could increase the permeability of TJs in early weaned piglets [41] and the probiotics mix of *B. licheniformis* and *B. subtilis* was proven to reduce ETEC F4-induced membrane barrier disruption by maintaining proper position of ZO-1 in weaned pigs [20]. These supported evidences were consistent with our presented data that ETEC F4 challenge could decrease the expression of ZO-1 and OCLN in IPEC-J2 cells, and these down-regulations could be partially reversed by the application of *B. licheniformis* PF9 (Fig. 5). Moreover, immunofluorescence staining showed that *B. licheniformis* PF9 promoted the expression of F-actin, ZO-1 and OCLN in the cytomembrane (Fig. 4). Furthermore, TEER is usually considered a good indicator to evaluate the integrity and tightness of intestinal epithelial barrier model [11, 42]. It was demonstrated that the relative TEER and FD4 values did significantly decrease and increase, respectively, after ETEC F4 stimulation in IPEC-J2. However, these damages could be prevented by the treatment of *B. licheniformis* PF9 to some degree (Fig. 6). As a whole, these results indicated that *B. licheniformis* PF9 could enhance barrier functions in the IPEC-J2 cells. Therefore, in our study, *B. licheniformis* PF9-enhanced expression of TJ proteins seem to contribute to strengthening the epithelial barriers against bacterial pathogen infections.

Quorum sensing (QS) is a bacterial cell-cell communication process, regulating the expression of genes that affect a variety of cellular processes including bioluminescence, sporulation, biofilm formation, antibiotic production, and virulence factors secretion [43]. QS is based on production, secretion and response to small signaling molecules, called autoinducers (AI) [44]. AI-2, a universal autoinducer, is widely distributed in numerous Gram-positive and Gram-negative bacteria [45]. LuxS plays an important role in AI-2 biosynthesis, involving conversion of ribose-homocysteine into homocysteine (SRH) and 4,5-dihydroxy-2,3pentanedione (DPD) [45, 46]. It was reported that *B. subtilis *R-18 could inhibit QS-mediated virulence in *Serratia marcescens* [47]. In the present study, *B. licheniformis* PF9 could down-regulate the expression of bacterial genes related to quorum sensing (*luxS*), as well as STs (*estA* and *estB*) and LT (*elt*) toxin secretion in ETEC F4-infected IPEC-J2 cells (Fig. 7). Therefore, *B. licheniformis* PF9 could be involved in alleviating ETEC F4-induced cytotoxicity by decreasing QS and virulence factors secretion in IPEC-J2 cells.

The earliest immune cells distinguishing self-molecules from extraneous structures is based on the conserved pathogen-associated molecular patterns (PAMPs) that are recognized by multiple classes of molecules called pattern recognition receptors (PRRs) [48]. Toll-like receptors (TLRs), a class of PRRs, are transmembrane proteins that are localized to the plasma or endosomal membranes of many types of cells. The activation of TLRs mediates pro-inflammatory signaling via the myeloid differentiation primary response protein (MYD88) and TIR domain-containing adapter molecule 1 (TRIF) pathways [49]. Research also found that TLR2/TLR4-mediated NF-κB signaling pathways have been involved in the ETEC-induced inflammation process in IPEC-J2 cells [50]. From our results, *B. licheniformis* PF9 could inhibit the inflammation process via TLR4-mediated NF-κB signaling pathways. Recently, links were established between inflammation and ETEC enterotoxins. A recent study has shown that LTs also can activate both NF-κB and MAPK signaling pathways, involved in ETEC adherence [51]. Most of the secreted LT is found to be associated with outer membrane vesicles (OMVs). LT-OMVs induce a great number of inflammatory cytokines as IL-6 and TNF-α through p38, ERK1/2 and NF-κB signaling pathways in T84 cells [52]. However, the relationship between enterotoxin and inflammation and its underlying mechanism on *B. licheniformis* PF9 resistance to ETEC infection need further research.

## Conclusions

In conclusion, our data nominated that *B. licheniformis* PF9 might inhibit TNF-α, IL-8, and IL-6 production due to the inhibition of NF-κB signaling pathway. It might also be possible for *B. licheniformis* PF9 to enhance the cell integrity according to the up-regulated expression of tight junction proteins. Our research provides data to understand that probiotics could improve gut health by improving their inflammatory and intestinal barrier during ETEC infection at the cellular level. Furthermore, the probiotic effects on pathogenic prevention in the animal model would be emphasized in the future.

## Supplementary Information

**Additional file 1: Table S1.** The primer sequences of the target genes and the internal reference gene used by qPCR in IPEC-J2 Cells . **Table S2.** The primer sequences of the virulence-related genes and the internal reference gene used by qPCR in ETEC F4.

## Data Availability

The data used to support the findings of this study are available from the corresponding author upon reasonable request.

## Abbreviations

ETEC: Enterotoxigenic *Escherichia coli*
*B. licheniformis*: *Bacillus licheniformis*
IPEC-J2: Intestinal porcine epithelial cell line
NF-κB: Nuclear factor-κB
TNF-α: Tumor necrosis factor-α
IL: Interleukin
TLR4: Toll-like receptor 4
ZO-1: Zona occludens 1
OCLN: Occluding
TEER: Trans-epithelial electrical resistance
IECs: Intestinal epithelial cells
TJs: Tight junctions
DMEM/F12: Dulbecco’s modified Eagle’s medium nutrient mix F12
FBS: Fetal bovine serum
EGF: Epidermal growth factor
TSB: Tryptic soy broth
CycA: Cyclophilin-A
gapA: Glyceraldehyde-3-phosphate dehydrogenase A
CLDN1: Claudin-1
CLDN3: Claudin-3
OD: Optical density
LDH: Lactate dehydrogenase
LTs: Labile toxins
STs: Stable toxins
QS: Quorum sensing
PAMPs: Pathogen-associated molecular patterns
PRRs: Pattern recognition receptors
TLRs: Toll-like receptors
TRIF: TIR domain-containing adapter molecule 1
OMVs: Outer membrane vesicles
