# Supplementary material for: Bacillus licheniformis PF9 improves barrier function and alleviates inflammatory responses against enterotoxigenic Escherichia coli F4 infection in the porcine intestinal epithelial cells
Source: J Anim Sci Biotechnol. 2022 Jul 8;13:86. doi: 10.1186/s40104-022-00746-8 (PMC9264548; doi:10.1186/s40104-022-00746-8)
Supplement: Supplementary file 1 — Additional file 1: Table S1. The primer sequences of the target genes and the internal reference gene used by qPCR in IPEC-J2 Cells . Table S2. The primer sequences of the virulence-related genes and the internal reference gene used by qPCR in ETEC F4. [file 40104_2022_746_MOESM1_ESM.docx]

| **Table S1** The primer sequences of the target genes and the internal reference gene | | | |
| --- | --- | --- | --- |
| **Gene name** | **Sequence (5′ to 3′)** | **Accession Number** | **Product Size, bp** |
| Cytokines genes |  |  |  |
| *TNF-α* | F: TTCCAGCTGGCCCCTTGAGC | NM_214022.1 | 146 |
|  | R: GAGGGCATTGGCATACCCAC |  |  |
| *IL-8* | F: AGAGGTCTGCCTGGACCCCA | NM_213867.1 | 126 |
|  | R: GGGAGCCACGGAGAATGGGT |  |  |
| *IL-6* | F: TTCACCTCTCCGGACAAAAC | NM_214399.1 | 122 |
|  | R: TCTGCCAGTACCTCCTTGCT |  |  |
| Barrier function genes |  |  |  |
| *ZO-1* | F: GATCCTGACCCGGTGTCTGA | XM_021098896.1 | 200 |
|  | R: TTGGTGGGTTTGGTGGGTT |  |  |
| *CLDN1* | F: CTGTGGATGTCCTGCGTGT | NM_001244539.1 | 93 |
|  | R: GGTTGCTTGCAAAGTGGTGTT |  |  |
| *CLDN3* | F: CTACGACCGCAAGGACTACG | NM_001160075.1 | 123 |
|  | R: TAGCATCTGGGTGGACTGGT |  |  |
| *OCLN* | F: GAGAGAGTGGACAGCCCCAT | NM_001163647.2 | 163 |
|  | R: TGCTGCTGTAATGAGGCTGC |  |  |
| Toll-like receptors |  |  |  |
| *TLR2* | F: ACGTATCCATCAATGAACACTGC | NM_213761.1 | 153 |
|  | R: GTCCGTTAAGGGTGCAGTCA |  |  |
| *TLR4* | F: GCCATCGCTGCTAACATCATC | NM_001113039 | 108 |
|  | R: CTCATACTCAAAGATACACCATCGG |  |  |
| *TLR5* | F: GTTCTTTATCCGGGTGACTT  R: AATAAGTCAGGATCGGGAGA | NM_001348771.1 | 86 |
| *TLR7* | F: GCTGTTCCCACTGTTTTGCC  R: ACTTGCGGTTGACTGAGGTT | NM_001097434.1 | 107 |
| NF-κB signaling pathway genes |  |  |  |
| *MYD88* | F: CCATTCGAGATGACCCCCTG | NM_001099923.1 | 183 |
|  | R: TAGCAATGGACCAGACGCAG |  |  |
| *IRAK1* | F: CAAGGCAGGTCAGGTTTCGT | XM_003135490.4 | 115 |
|  | R: TTCGTGGGGCGTGTAGTGT |  |  |
| *TRAF6* | F: CAAGAGAATACCCAGTCGCACA | NM_001105286.1 | 122 |
|  | R: ATCCGAGACAAAGGGGAAGAA |  |  |
| *TAK1* | F: GCCACCGTAAAACTGCTTCAT | NM_001114280.1 | 196 |
|  | R: GCTGGCTTTTCTGAGGTTGG |  |  |
| Internal reference gene |  |  |  |
| *CYCA* | F: GCGTCTCCTTCGAGCTGTT | NM_214353.1 | 160 |
|  | R: CCATTATGGCGTGTGAAGTC |  |  |

Note: *TNF-α*: tumor necrosis factor α; *IL-6* and *8*: interleukin 6 and 8; *ZO-1*: zonula occludens-1; *CLDN1* and *3*: claudin 1 and 3; *OCLN*: occludin; *TLR2*, *4*, *5*, and *7*: toll-like receptor 2, 4, 5, and 7; *MYD88*: myeloid differentiation primary response protein; *IRAK1*: IL-1R-associated kinase 1; *TRAF6*: TNF receptor-associated factor 6; *TAK1*: TGFβ-activated kinase 1; *CYCA*: Cyclophilin-A.

**Table S2** The primer sequences of the virulence-related genes and the internal reference gene used by qPCR in ETEC F4

| **Gene name** | **Sequence (5′ to 3′)** |
| --- | --- |
| Virulence-related genes |  |
| *luxS* | F: ATGCCGTTGTTAGATAGCTTCAC |
|  | R: CTAGATGTGCAGTTCCTGCAACT |
| *FaeG* | F: ACTGGTGATTTCAATGGTTCG |
|  | R: GTTACTGGCGTAGCAAATGC |
| *estA* | F: CAACTGAATCACTTGACTCTT |
|  | R: TTAATAACATCCAGCACAGG |
| *estB* | F: TGCCTATGCATCTACACAAT |
|  | R: CTCCAGCAGTACCATCTCTA |
| *elt* | F: TCTCTATGTGCATACGGAGC |
|  | R: CCATACTGATTGCCGCAAT |
| Internal reference gene |  |
| *GAPA* | F: TCCGTGCTGCTCAGAAACG |
|  | R: CACTTTCTTCGCACCAGCG |
